# Phrase-Level Modeling of Expression in Violin Performances

**DOI:** 10.3389/fpsyg.2019.00776

**Published:** 2019-04-11

**Authors:** Fábio J. M. Ortega, Sergio I. Giraldo, Alfonso Perez, Rafael Ramírez

**Affiliations:** Music Technology Group, Machine Learning and Music Lab, Department of Communication and Information Technology, Pompeu Fabra University, Barcelona, Spain

**Keywords:** expressive music performance, machine learning, music information retrieval, violin, music pedagogy

## Abstract

**Background:** Expression is a key skill in music performance, and one that is difficult to address in music lessons. Computational models that learn from expert performances can help providing suggestions and feedback to students.

**Aim:** We propose and analyze an approach to modeling variations in dynamics and note onset timing for solo violin pieces with the purpose of facilitating expressive performance learning in new pieces, for which no reference performance is available.

**Method:** The method generates phrase–level predictions based on musical score information on the assumption that expressiveness is idiomatic, and thus influenced by similar–sounding melodies. Predictions were evaluated numerically using three different datasets and against note–level machine–learning models, and also perceptually by listeners, who were presented to synthesized versions of musical excerpts, and asked to choose the most human–sounding one. Some of the presented excerpts were synthesized to reflect the variations in dynamics and timing predicted by the model, whereas others were shaped to reflect the dynamics and timing of an actual expert performance, and a third group was presented with no expressive variations.

**Results:** surprisingly, none of the three synthesized versions was consistently selected as human–like nor preferred with statistical significance by listeners. Possible interpretations of these results include the fact that the melodies might have been impossible to interpret outside their musical context, or that expressive features that were left out of the modeling such as note articulation and vibrato are, in fact, essential to the perception of expression in violin performance. Positive feedback by some listeners toward the modeled melodies in a blind setting indicate that the modeling approach was capable of generating appropriate renditions at least for a subset of the data. Numerically, performance in phrase–level suffers a small degradation if compared to note–level, but produces predictions easier to interpret visually, thus more useful in a pedagogical setting.

## 1. Introduction

When practicing an instrument on their own, music students focus mostly on playing correctly, and not enough on playing expressively. Software tools for assisting practice could address this issue by offering suggestions and feedback about expressive aspects of performance such as dynamics, articulation, and timbre quality, ensuring students keep these concerns in mind at all times. However, the implementation of this feature requires equipping software tools with models of performance that enable them to generate coherent suggestions for any musical piece included in the practice routine. This work explores the feasibility of that approach by proposing such a model and analyzing its suggested variations in dynamics and note onset timing of solo violin pieces.

Much of the work in the field of expressive performance modeling deals with predicting expressive features of notes in isolation, however, in this particular scenario where the goal is providing guidance to a human player, long–term movements and character of expression take preference over minute variations that occur note–by–note. For that reason, the devised model bases its outputs on features of compositions that play a strong role in representing musical context: *phrasing* and *melody*.

*Phrasing* is defined as the separation of successive notes from one another, singly or in groups, by a performer, and the manner in which this is done (Chew, 2001). In the context of this work, it refers to the separation of sequences of notes into the smallest cohesive groups typically acknowledged in the western musical canon, the *motifs* (Drabkin, 2001), though we refer to such groups as *motifs* or *phrases* interchangeably.

As for *melody*, it is understood for our purposes as a pattern characterized by the relationships of pitch and duration of notes in a phrase, that is, the tones of a phrase itself along with its rhythmic qualities, but isolated from harmonic context.

Expression in music performance has been actively studied for over two decades from several standpoints. Many authors dedicate their efforts to computationally modeling expressive performance actions as surveyed by Kirke and Miranda (2009) and Widmer and Goebl (2004) allowing a range of applications, from automatic performer recognition (Molina-Solana et al., 2008) to adding realism to automatic performance and improvisation (Grachten, 2001; Widmer et al., 2009). Other notable aspects of expression in music include representation and communication of emotion (Juslin and Timmers, 2010) and the role of motion (Meyer, 1956; Huberth and Fujioka, 2018) and expectations (Huron, 2006; Pearce et al., 2010), as summarized by Juslin (2003). The problem of teaching expressive performance in an effective manner has also been studied, though to a lesser extent. Woody (2006) investigates different methods used by teachers to communicate the desired expressive intentions and their effectiveness. Some intersection between performance modeling and pedagogy have been explored by Juslin et al. (2006) with a system trained to give feedback to students on their ability to communicate specific emotions through music in the guitar. The pianoFORTE system (Smoliar et al., 1995) for assisting piano instructors in discussing and instructing expressiveness also serves as a seminal reference to this work.

Existing approaches to performance modeling have mostly been developed for piano (Kirke and Miranda, 2013). Though partially applicable, the many other expressive possibilities available to violin performers justify developing more specific models. From the existing piano approaches we highlight the DISTALL system (Tobudic and Widmer, 2003) which also produces timing and dynamics predictions incorporating phrase–level analyses.

Specifically targeting the violin, the works of Maestre (2009) and Van Der Linden et al. (2011) share a similar goal to ours, the first one by predicting bow movement from score notation and the second by seeking to correct violin students' posture using vibrotactile feedback in wearable devices. Finally, Ramirez et al. (2013) provide a framework for expressive feature prediction in violin performances, albeit in terms of classification rather than regression as we do.

Evaluating expression in violin performances presents several distinct challenges when contrasted with piano performances. The advantage of the piano lies in the fact that the interaction between performer and instrument can be summarized with only a few parameters (mainly instants and velocities of keys and pedals pressure and release). Thus, for piano, modeling expression in these parameters is often sufficient for synthesizing convincing performances. Since the violin offers musicians several other expressive dimensions (e.g., attack speed, vibrato, intonation, etc.), our approach to validating the restricted set of modeled features—timing and dynamics—has been to ensure that the synthesized sounds lacked any other type of expressive variation. However, in many situations, these variations work in conjunction and justify one another.

The model as implemented forms expressive suggestions based on information taken from the musical score only, with the purpose of presenting them to students as either visual feedback, such as an orchestra conductor does, or by overlaying graphs on the score itself, or auditory, by providing synthesized examples or even accompaniment. At this stage, however, the mode output is raw, and has been analyzed in terms of similarity to an actual expert performance and perceived human-likeness, essentially exploring whether an automatic recognition of phrasing and melody from a score can used as predictor of its performance attributes. Consequently, this is an indirect way of addressing the broader question: to what extent do these musical structures influence performance?

## 2. Proposed Model

As previously mentioned, the developed algorithm models the dynamics and timing for a given musical piece, meaning that given the computed score features, it outputs recommended loudness levels for its performance, and indications of when to rush or drag the tempo. Since its intended application is as a pedagogical aid, we are interested in modeling the long–term variations in expression that arise from the players' interpretation of the music rather than short–term variations that are not only impractical to communicate in real time but are also, to a large extent, influenced by the same long–term intentions as well as some unconscious factors such as playing technique. For that reason the model is trained on phrases rather than notes. Nevertheless, it is built on top of a note–level structure, which means all training data are initially processed in the form of note–level inputs and outputs, and later aggregated into phrase features as informed by a phrase boundaries determination method.

The note–level input features of interest are note pitch and note duration. These were extracted from musicXML by a parser written in MATLAB (code available in GitHub^1^). Other features present in scores such as note articulation, dynamics annotations, and song key were also extracted but were left out of the modeling in an effort to explore exclusively the effect of melodic and rhythmic content in expression. Also, by using only pitch and duration, the model is readily applicable to even the simplest music notation such as MIDI, allowing us to expand our training set and to make use of automatic transcriptions in future iterations.

The note–level output features are the mean level of loudness at which each note was performed in the recordings, and their onset times and durations. To compute them, the audio files were normalized to −0.1 dB and then input in the Tony software (Mauch et al., 2015), where the onsets and offsets played were recognized by a hidden Markov model designed by Mauch et al. over the pYin (Mauch and Dixon, 2014) algorithm and manually corrected. This information about onsets and offsets could then be exported to a table and their corresponding pitches matched against the pitches from score notes using an implementation of the Needleman–Wunsch algorithm for optimal matching of sequences (Needleman and Wunsch, 1970), thus remedying discrepancies between score and performance (e.g., grace notes). Finally, the mean loudness level *v* of each note could be computed according to Equation 1, where *s* are audio samples in the time-frame of note *n*.

(1) $$v_{n}=20\;\cdot \;\log\left(\sqrt{\sum_{i={\text{onset}}_{n}}^{{\text{offset}}_{n}}s_{i}^{2}}\right)$$

**Figure 1:**
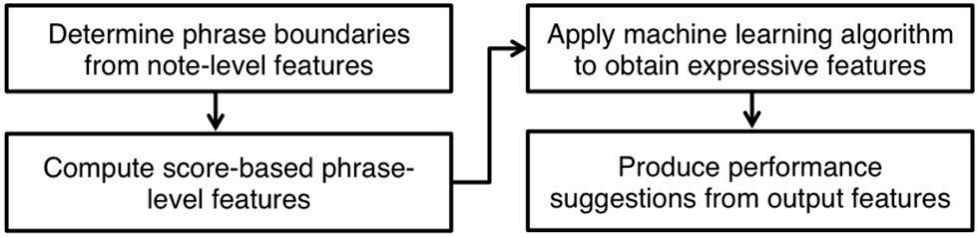
Summary of the steps for producing suggestions of expression.

The flowchart in Figure 1 outlines the steps involved in computing the expressive features of a piece with the proposed model. A detailed description of each step follows.

The automatic detection of melodically–coherent phrases uses a top-down approach based on the Local Boundary Detection Model (LBDM) (Cambouropoulos, 2001). The LBDM outputs a value for each note in a piece which represents the likelihood of that note being a local boundary (i.e., the first in a phrase) taking into account the variations in pitch, note durations and presence of rests. The segmentation algorithm uses these values for recursively evaluating whether a segment should be split further. The following pseudocode illustrates how that is achieved:

**Algorithm 1:** Piece Segmentation

** Input:** The LBDM value *l*_*i*_ for every note *i* = 1..*end*.
1: **procedure** Segment([*l*_*k*_, *l*_*k*+1_, …, *l*_*n*_])
2: **if** *n* − *k* ≤ 10 **then**
3: **return** one segment, from *k* to *n*
4: **else**
5: Calculate z-scores from values [*l*_*k*+2_, …, *l*_*n*−1_]
6: **if** the largest z-score *z*(*l*_*max*_)> 2 **then**
7: **return** Segment([*l*_*k*_, …, *l*_*max*−1_]), Segment([*l*_*max*_, …, *l*_*n*_])
8: **else**
9: **return** one segment, from *k* to *n*

As a result of its structure, the algorithm gravitates toward phrases of approximately 10 notes without imposing a hard restriction. Even though phrases of a single note might be musicologically acceptable, we intentionally prevent their occurrence since pieces with ambiguous phrase boundaries often cause the LBDM to output high likelihood values for consecutive notes in situations where one-note phrases would not be reasonable.

Once a piece is split into phrases, each phrase must be associated with a set of characteristics—phrase input features—that allow us to describe its expressive character as phrase output features.

The input features are essentially the key–invariant melodic contour, and the tempo–invariant rhythmic contour. The melodic contour is a time–series with one data point per phrase note beginning in zero and with each subsequent value equal to the difference in semitones between consecutive note pitches. As an example, a major triad in root position would be encoded as the sequence: 0, 4, 3. The rhythmic contour is also a time–series with one data point per phrase note; its first value is one and the others are equal to the ratio between each note in the phrase and the previous one.

The intelligence built into the model follows the principle that similar–sounding melodies are more likely to be performed similarly. In practice, this is implemented by applying a k-NN algorithm that, for each input phrase, locates its most similar phrases in the training set with respect to the described features. The measure applied for determining the degree of similarity between two phrases is an implementation of the method proposed by Stammen and Pennycook (1993), which itself is a dynamic time warping algorithm that interprets each phrase as a two-dimensional time series, the dimensions being our phrase input features. The cost function is based on euclidean distance, that is, the sum of squared differences between values in each dimension. The algorithm outputs a *distance* value which is the minimum cost required to warp the first time-series into the second, therefore, zero for equal melodies and a positive real number otherwise. The intuitive interpretation of the method is that two melodies are rated as more distant, or dissimilar, the more notes one has to include or change—in pitch, duration, or both—in order to make both melodies sound the same.

The phrase output features must describe the dynamics and timing of a phrase in terms that can be transposed to other pieces and contexts. Our implementation defines four of them: mean dynamic level, dynamic range, dynamic contour, and local tempo curve. These features are computed for every phrase in the training set recordings and act as references for the model's operation. When suggesting how to express a new phrase, all four features are decided by the algorithm for the new phrase using the available references.

The formal definition of each of these features depends on some metrics related to the entire piece. In a piece with *n* notes, each with loudness *v* (defined in Equation 1) and duration *d*, the mean loudness level *L* of the piece and its dynamic range *R* are:

(2) $$L=\frac{\sum_{i=1}^{n}v_{i}\;\cdot \;d_{i}}{\sum_{i=1}^{n}d_{i}}\;R={\text{max}}_{i}(v_{i})-{\text{min}}_{i}(v_{i})$$

Essentially, *L* is the weighted mean of loudness values *v*_*i*_ with respect to the durations *d*_*i*_, and *R* is the range of excursion of the same random variable.

Using these metrics, we define the mean dynamic level ℓ_*p*_ of a phrase *p* having *m* notes and beginning in note *k* as:

(3) $$\ell_{p}=\frac{1}{R}\;\cdot \;\left(\frac{\sum_{i=k}^{k+m}v_{i}\;\cdot \;d_{i}}{\sum_{i=k}^{k+m}d_{i}}-L\right)$$

This feature (ℓ_*p*_) refers to the character of a phrase (e.g., *forte*, pianissimo, etc.) and is represented by a single real number that measures how the loudness in the phrase deviates from the mean loudness in the piece, normalized by that piece's dynamic range.

The second output feature is the phrase dynamic range *r*_*p*_:

(4) $$r_{p}=\frac{1}{R}\;\cdot \;\left({\text{max}}_{p}(v_{p})-{\text{min}}_{p}(v_{p})\right)$$

Analogously to what happens between ℓ_*p*_ and *L*, output feature *r*_*p*_ is the phrase–level counterpart of *R*, and it measures the dynamic range of a phrase in “piece range” units.

Lastly, phrase contour (*C*_*p*_) is a function which describes how each note in a phrase *p* contributes to its dynamics once the effects of ℓ_*p*_ and *r*_*p*_ are discounted. Therefore, if ℓ_*p*_, *r*_*p*_, and *C*_*p*_ are known for *p*, the loudness value for each note *i*∈*p* can be determined by:

(5) $$v_{i}=L+R\;\cdot \;\left(\ell_{p}+r_{p}\;\cdot \;C_{p}(i)\right)$$

This definition has two implications. The first is that we can determine the values of *C*_*p*_ in each note *i* for phrases in the training set since their loudness values *v*_*i*_ are known. Using those values, *C*_*p*_ is fitted in a quadratic polynomial using the least–squares method, as inspired by observations regarding typical phrasing dynamics by McAngus Todd (1992) and other researchers (Gabrielsson et al., 1983; Tobudic and Widmer, 2003; Livingstone et al., 2010) who all point to their parabolic contour. This allows us to define *C*_*p*_ for all *p* in the training set using only three coefficients for each.

The second implication of Equation 5 is that determining ℓ_*p*_, *r*_*p*_, and *C*_*p*_ for a phrase *p* for which we wish to provide dynamics suggestions is enough to compute suggested loudness values *v*_*i*_ for all notes *i*∈*p*, as long as chosen values for *L* and *R* are provided. In practice, *L* represents the overall character of the piece and *R* its overall dynamic range, to which all phrases should conform. These can be set to default values or adapted according to a wider context (e.g., a lower *L* for an *adagio* than for an *allegro*). This aligns with our initial desire of characterizing phrases independently of context, so that the knowledge–base of the training set is applicable across all musical intentions.

Finally, the local tempo curve is defined as the function that describes how the tempo changes throughout the phrase. For each note *i*, its local tempo value *t*_*i*_ is computed as:

(6) $$t_{i}=\frac{60}{T}\;\cdot \;\frac{b_{i}}{{\text{ioi}}_{i}}$$

Where *T* is the piece tempo in beats per minute, *b*_*i*_ is the duration of note *i* in beats according to its rhythmic figure in the score, and ioi_*i*_ is the inter–onset interval between notes *i* and *i*+1. The local tempo curve of each phrase is, once again, the quadratic polynomial that best fits its local tempo values, for x–axis values spaced proportionally to *b*_*i*_.

For a suggested local tempo curve τ, one can use Equation 6 to compute the IOI of each note, since *t*_*i*_ is given by τ(*b*_*i*_) and the desired piece tempo *T* should be provided. Assuming that the first note starts at 0s and working sequentially, this defines the onset times of all notes.

It should be highlighted that the only processing step in the modeling that required manual intervention was the onset detection for note alignment. However, since this task was partially automated and completed without making use of score information, we are confident that the entire modeling process could be done automatically to satisfactory results, enabling its application in the desired pedagogical context.

## 3. Numerical and Perceptual Evaluations of the Proposed Model

### 3.1. Materials and Methods

The evaluation is divided into numeric and perceptual analyses. The numeric analysis checks if the score–related metrics of phrasing and melody correlate with the dynamics and timing of a performance whereas the perceptual analysis verifies if synthesized performances based on modeled expression possess human–like qualities.

Eight short (approximately 50 s each) musical excerpts were recorded by a professional violinist to be used for both model generation and evaluation, making up dataset *DS1*. The pieces were chosen from the violinist's repertoire with the intention of providing a wide range of moods and melodies of western classical violin, and were played solo and without metronome. The audio was captured from a single condenser microphone placed at close distance from the violin body and the scores of all recorded excerpts were manually transcribed into MusicXML^2^ format using the MuseScore software^3^. Though small for a typical machine–learning application, this dataset emulates an envisioned scenario where a student is interested in being presented with performance suggestions resembling a particular style or musician as represented by a short number of sample recordings. To enable such a use–case, it is important to verify whether our modeling strategy is sufficiently robust to provide meaningful results under those restrictions.

In the numeric analysis, the suggested loudness values computed from Equation 5 were interpreted as estimations and compared against the measured values in the recorded performances of the pieces. Likewise, the suggested onset times were compared to the performed ones for each piece. Test sets were built in a leave–one–out approach, meaning that dynamics and timing suggestions were generated for each available piece using the other seven recorded pieces as training set.

For the perceptual analysis, the note loudness values obtained from the model were converted into MIDI velocity values used to control the dynamics of synthesized versions of the pieces. The syntheses were made using Apple Logic Pro X's EXS24 sampler^4^, with violin samples obtained from the Freesound database (Akkermans et al., 2011). The sample set was chosen for its lack of vibrato, in order to minimize the influence of this other expressive element in the evaluation of the synthesized performances ^5^. The conversion of loudness values from dBFS (decibels relative to full-scale) to the MIDI velocity scale (1–128) followed the findings of Dannenberg (2006) who observed a square-law between velocity values and RMS amplitude in synthesized audio. Our mapping was empirically adjusted so that the dynamic range of pieces synthesized using velocities calculated from the amplitude of the recordings matched the dynamic range of the original audio of the same recordings.

Three versions of each piece were synthesized for the evaluation, the only difference being the supplied velocity values and note onset times and durations, resulting in different dynamics and timing for each of them: one version used velocity and onset values derived from the model suggestions as described above; a second version corresponds to the expression of the performer in the recordings, as measured for usage in the training set. The third and last version serves as baseline and scientific control, and uses the same velocity for all notes, its value being the mean value used in the “human” version to minimize discrepancies in volume level, and its timing has no fluctuation, the tempo being set to the mean tempo from the “human” version. Each of the three versions of the original 8 pieces were manually divided into 3 excerpts of approximately 15 s each and their audio normalized (applying the same gain to all three versions of an excerpt to prevent from modifying their relative dynamic range). Finally, the eight most complete, melodic sounding of those 24 excerpts were selected for the evaluation.

The evaluation was conducted by means of an on-line survey. Participants were instructed to hear randomized pairs of audio samples from the synthesized pieces, always consisting of two out the three existing versions of an excerpt. They were then presented with two questions for which to choose between audio samples 1 or 2: “In terms of dynamics (the intensity of volume with which notes are expressed), select which audio sample sounds most like a human performance to you.” and “Which performance did you like best?” Finally, participants were instructed to answer, from 1 to 5, “How clearly do you perceive the distinction between the two audio samples?” A space for free comments was also included in each screen to encourage participants to share insights about their thought–process.

**Figure 2:**
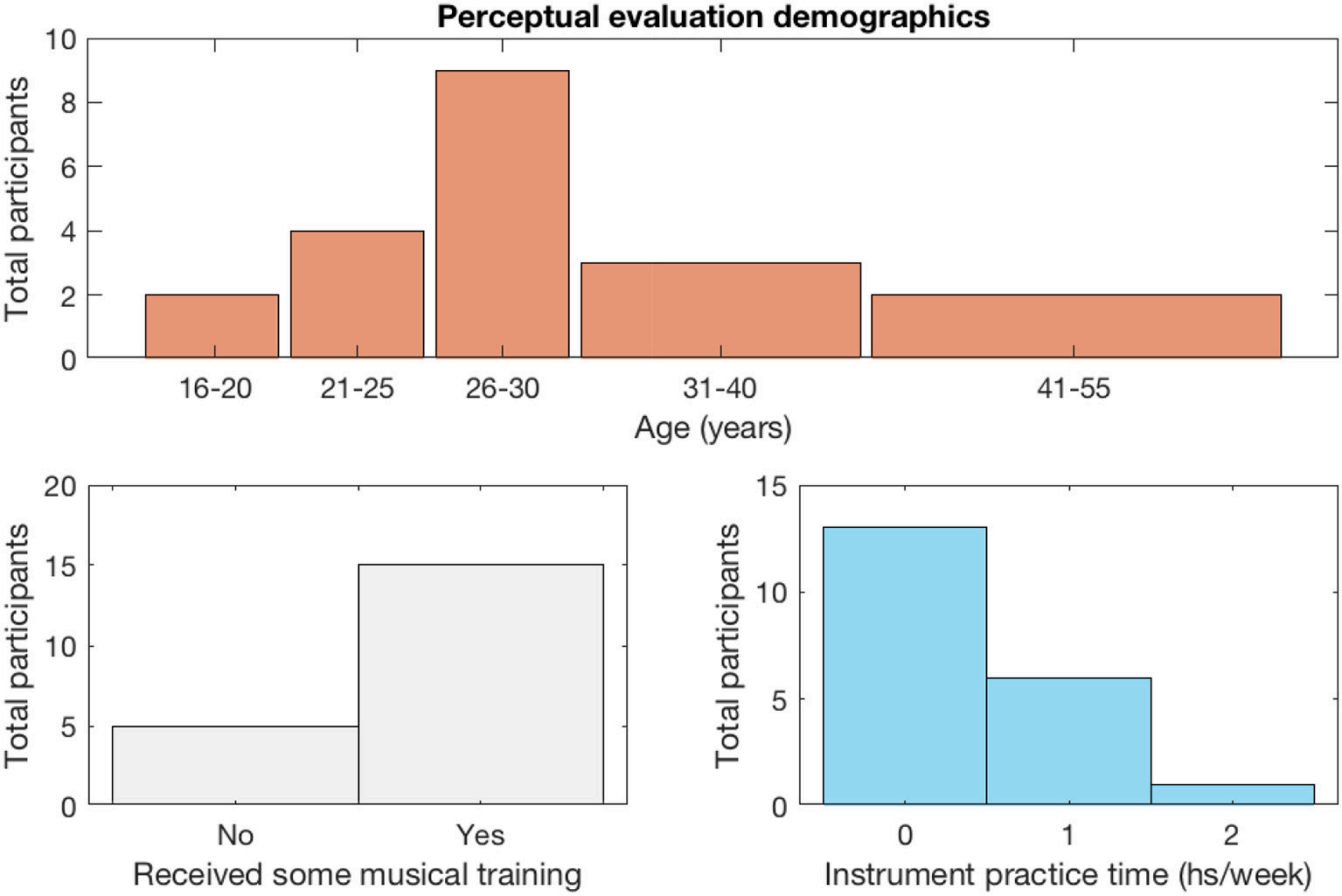
Summary of perceptual evaluation participants information.

A total of 20 people participated in the experiment. Recruitment was carried out by personal invitation and each participant was assisted in accessing the web page containing the survey and its instructions using their own computers and audio equipment. Each of them was asked to provide answers to 16 pairs of melodies as described above, but early abandonment was allowed. This provided a total of 305 pairwise comparisons. Figure 2 shows a breakdown of the profile of participants in terms of age and musical training.

### 3.2. Results

**Figure 3:**
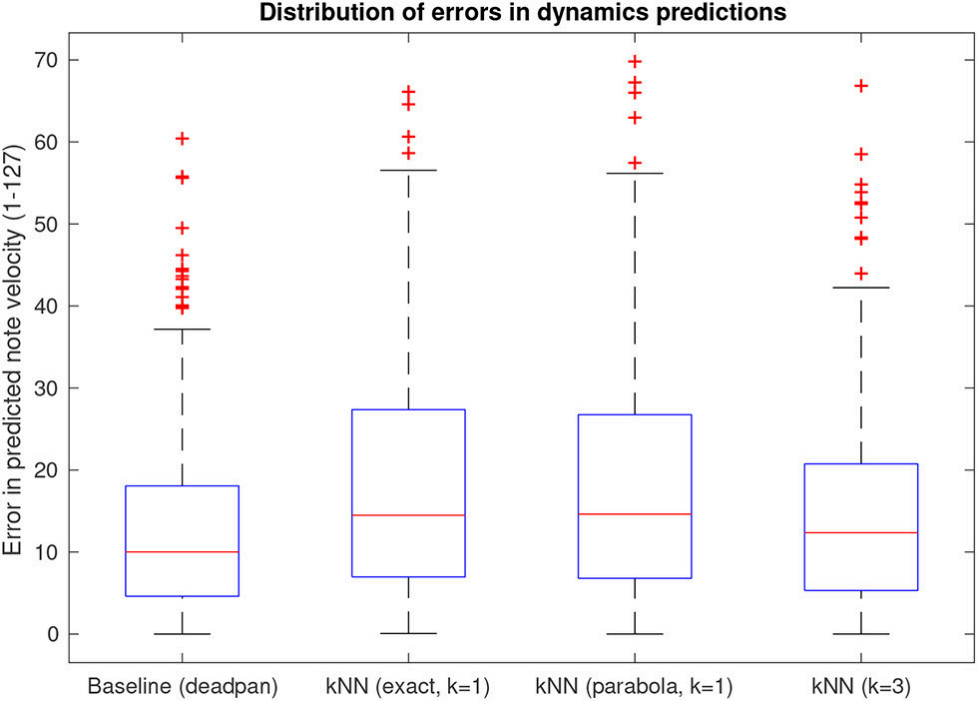
Aggregate mean absolute errors when considering dynamics suggestions as performance predictors.

Numeric analysis results are presented in Figure 3, which shows distributions of mean absolute errors in dynamics predictions measured on note level and aggregated across all modeled pieces. In this case, model outputs are interpreted as predictions of dynamics and the difference between predicted values for each note and the measured values in an actual performance make up the boxplot values. The baseline values measure the mean absolute difference between the dynamics of each performed note and the mean dynamics of the piece, therefore, it represents the lowest possible errors for a prediction with no dynamic variation.

Boxplot “kNN (exact, *k* = 1)” is the result of modeling the phrase contour function (*C*_*p*_ in the model) as a piecewise linear function containing data points from all notes in the reference phrase whereas boxplot “kNN (parabola, *k* =1)” defines phrase contour as a quadratic function such as explained in the previous section. Their similar error profiles indicate that the simplified parabolic representation retains all relevant information validating its use. Errors in predictions of timing deviations fared similarly in comparison with a “deadpan” (inexpressive) baseline showing no onset time deviation.

**Figure 4:**
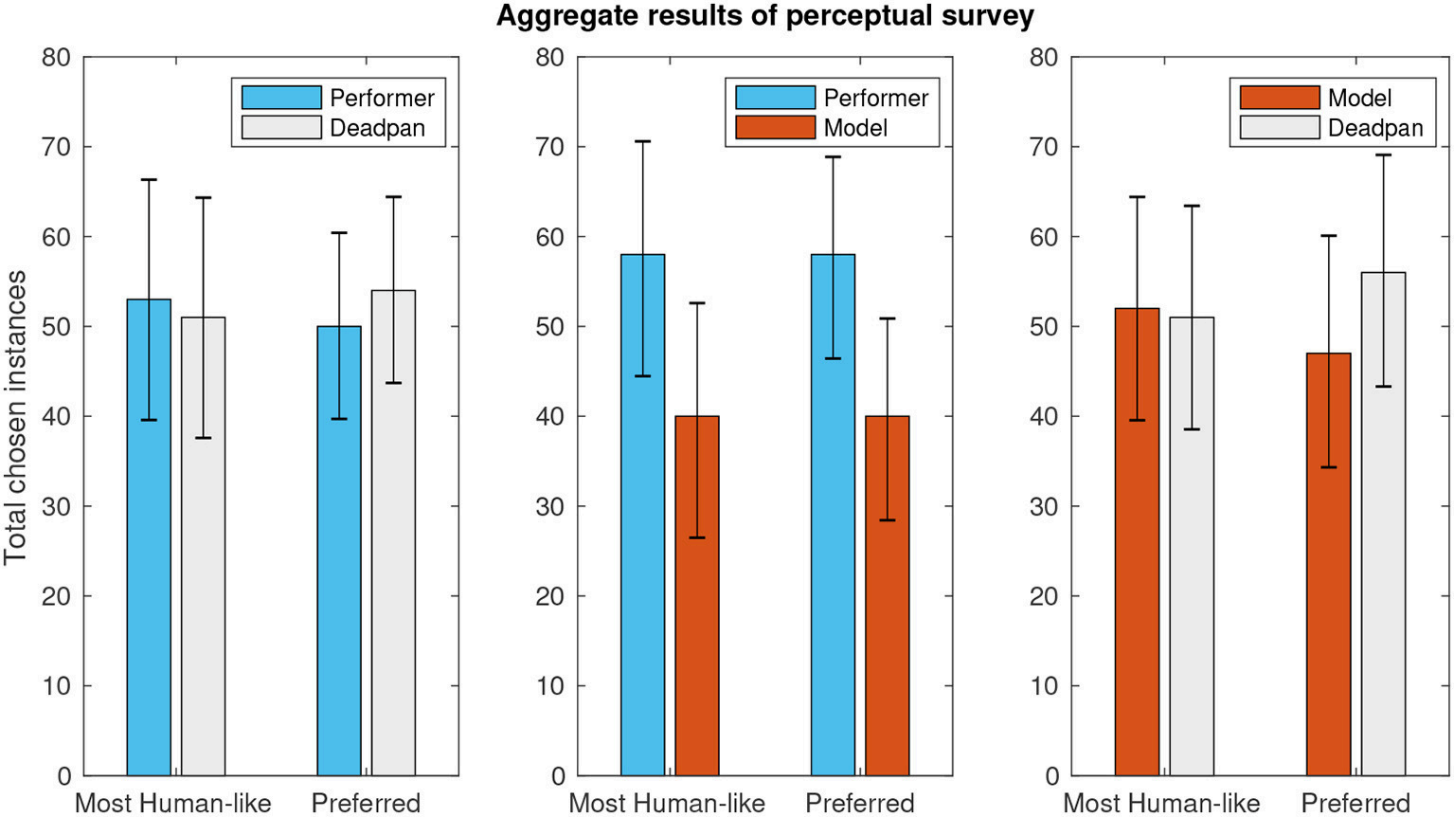
Aggregate results of perceptual survey pairwise comparisons.

In the perceptual analysis, a total of 305 pairs of melodies were compared by listeners in terms of human–likeness and personal preference. The mean perceived distinction between pairs was 3.41 ± 0.13 (on a 1–5 scale, α = 0.05). Figure 4 shows the results divided into the three possible pairs according to expressive character: (C1) choice of human–based expression over “deadpan” baseline, (C2) human–based over modeled expression, and (C3) modeled expression over deadpan. A sign–test with confidence–level of 95% controlled for 5% false discovery rate using the Benjamini-Hochberg method fails to reject the null hypothesis in all comparisons (*p*-values listed in Table 1), thus indicating that none of the versions was perceived as significantly more human–like nor preferred by users consistently.

**Table 1:** Measured *p*-values for all perceptually evaluated comparisons.

**Comparison**	**Question**	***p*-value**
C1	Human–likeness	0.7500
	Preference	0.8016
C2	Human–likeness	0.1440
	Preference	0.1440
C3	Human–likeness	0.7500
	Preference	0.8378

**Figure 5:**
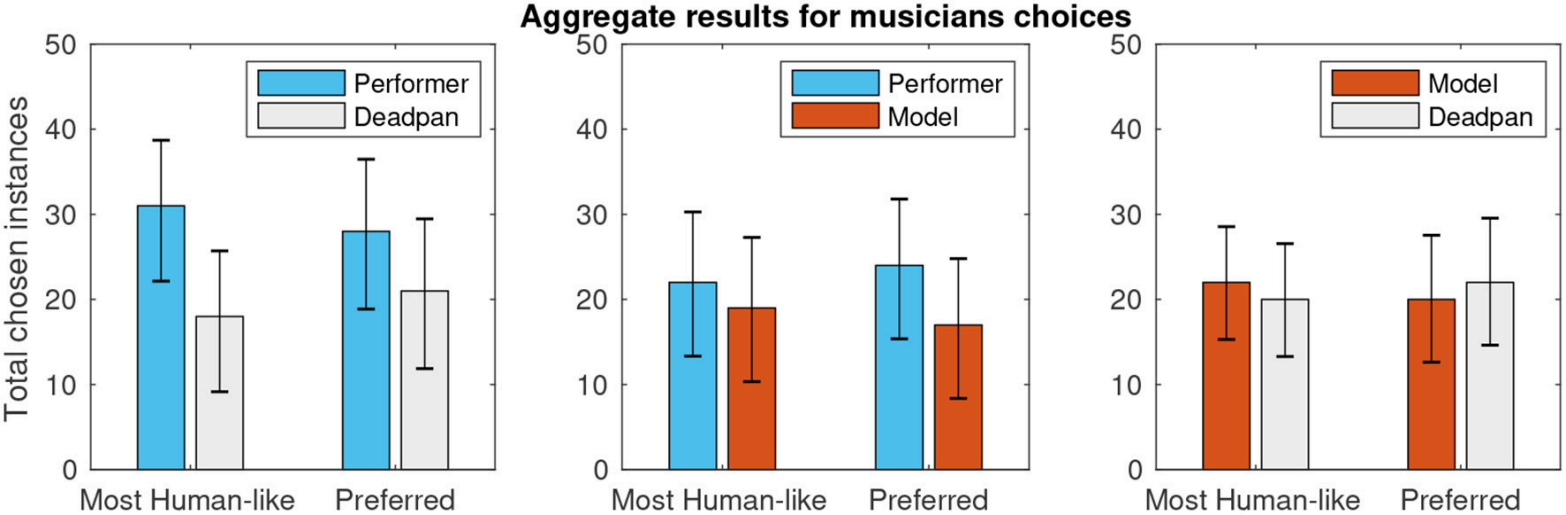
Subset of perceptual survey results for musically active participants.

Lastly, Figure 5 helps to provide some insight about the test setup by showing aggregate results of all comparison classes when considering exclusively the responses given by musically active participants of the survey, here characterized by the subset of people who reported practicing an instrument for at least one weekly hour.

### 3.3. Discussion

The large variance observed for the boxplots of model errors in the numeric analysis indicate that predictions for some sections of the pieces share similar dynamics to those performed in the reference recordings whereas other sections differ. Increasing the number of neighbors considered from one to three is effective at pruning out eccentric predictions as can be seen by the shorter tail of the distribution, but has been observed to also reduce the overall dynamic range of the output, making renditions a bit “dull.” In fact, this effect is expected for a small dataset such as DS1, since there ought to be very few examples of sufficiently similar melodies to be selected as nearest neighbors. Consequently, in such conditions, employing a single nearest neighbor is the most promising approach for a perceptually valid output, since the most melodically–similar phrase represents the available data point most likely to have applicable expression data for a given target melody, and copying such data parameters from the same sample retains the coherence between different expressive output variables. For this reason, and considering the success of the parabolic representation as a parametric model of contour indicated by their similar error distributions, the model with *k* = 1 and parabolic phrase contour was used for the synthesis of modeled performances for the perceptual evaluation.

The overall higher median errors observed in all measurements indicate that with DS1 as training set the model is not accurate at predicting timing and dynamics, but since there is no single correct interpretation of a musical piece, this result is not enough to dismiss the model as a tool for suggestions of expression, hence the utility of the perceptual validation.

Regarding perceptual analysis results, typically (Katayose et al., 2012; Bresin and Friberg, 2013), one would expect a wide dominance of human–based renditions over inexpressive ones, which was not verified in the results of C1. The inclusion of such cases in the survey was intended as a mechanism for validating the experimental setup, since the corpus of existing results in this field has been based mostly on piano works, and, to our knowledge, no similar setup has been investigated for violin pieces. The absence of a clear tendency toward the human–based synthetic performance prevents us from reaching a definitive conclusion regarding the effectiveness of the model until the causes for these unexpected results can be verified.

A deeper investigation of the results does offer fruitful insights, however. Ratings for the measure of perceived distinction between audio clips was generally high across all comparisons. For C1, in particular, its mean value was 3.31 and standard error, 0.12 (on a scale of 1–5). This strongly suggests that participants were able to perceive differences in the renditions, but still reached conflicting decisions. Reflecting upon this fact and contrasting the melodies present in our dataset against pieces typically found in benchmark datasets (e.g., as used by Oore et al., 2018) point to two main causes for participant disagreements: (1) the lack of expressive features such as variable attack–time, vibrato, and timbre variations, which often work in conjunction with the modeled features, facilitating their interpretation; and (2) the use of pieces written for an ensemble (namely violin and piano) without the accompanying instruments, which removes the melodies from their contexts and also limits the range for expression because of the requirement of musician synchronization.

This view is reinforced by some participant comments. One of them states, after declaring preference for the deadpan rendering over the human–based one: “*Little big ambiguous; A is more flat and regular, but it kind of depends on the context whether this would be appropriate or not.”* A, in that case, being the deadpan performance. Another one commented: “*I prefer the dynamics in B and the time in A. It's easy to distinguish them, but no one sound more human than the other.”(sic)*. In this case, B was a human–based rendering, and A, a deadpan one. Some comments can also be found which favor the modeled rendering, e.g., “*I prefer the dynamics in A and the time fluctuation in B.”* for a comparison where A corresponds to the modeled rendering, and B, to the human–based rendering.

From the musicians' results graph, it can be seen that the percentage of choices favoring the deadpan renditions is smaller in this subset than in the full result set, which could reflect a higher ability of the musicians in interpreting the performances even out of context. Furthermore, what is encouraging in the musicians' data is that the percentage of choices favoring the modeled performance is larger than in the full set, which is a hint of evidence in the direction of our own perception that the proposed modeling approach can yield convincing results under some conditions.

## 4. Further Numerical Evaluations With Augmented and New Datasets

### 4.1. Materials and Methods

For further analysis, the previously presented dataset (DS1) was complemented by the recordings of the first violin from the String Quartet number 4, *opus* 18 by Ludwig van Beethoven contained in the public dataset collected by Marchini et al. (2014) as part of their study on ensemble performance. As in the previous setting, the scores were manually transcribed into MusicXML format using the MuseScore software. The super set containing recordings from both sources constitutes dataset *DS2* and includes a total of 13 min of audio.

Finally, for a look at the model's performance under a large collection of data, a random sample of approximately 1 h of audio was taken from the 2017 recordings in the Maestro dataset (Hawthorne et al., 2018) even though it consists entirely of piano pieces. We refer to this collection as DS3. The summary of each dataset's characteristics is shown in Table 2.

**Table 2:** Summary of all datasets used.

**Name**	**Instrument**	**Total time**	**Num. instances**	**Notation**
DS1	Violin	6′43″	68	MusicXML
DS2	Violin	13′01″	192	MusicXML
DS3	Piano	57′08″	2706	MIDI

For ensuring consistently–sized test sets in spite of a larger variance in piece durations, all testings in both DS2 and DS3 were effectively 10–fold cross–validations on phrases, that is, datasets were split into 10 subsets by random sampling phrases without repetition, and each of those was used as test set in a different round. As in the previous scenario, note–level mean absolute errors between performance and model predictions were the chosen metric for all modeled expressive features.

To observe how the proposed modeling approach fares against more conventional models that rely on note features rather than phrase features, we computed 41 note features from score information and derived musicological inferences using dataset DS2, and employed the resulting feature vectors for predicting note velocity values and local tempi using various algorithms as implemented in the Weka machine learning software tool, version 3.8.3 (Frank et al., 2016). Code for reproducing the feature extraction and model building steps is available at a public Github repository^6^.

As an exploration of the impact of larger datasets in the model performance, *motifs* from DS3 were uniformly sampled without replacement into an increasingly larger subset. This growing subset of DS3 was used for model training, and mean absolute errors in dynamics predictions were recorded for tests using cross–validation as well as the training sets themselves with various subset sizes up to the size of the full DS3, and varying parameter *k* in the k-nearest neighbors from 2 to 8.

### 4.2. Results

Table 3 summarizes the results for the different note–level models implemented in Weka as well as the proposed phrase–level model using DS2: the second and third columns indicate Pearson's correlation and mean absolute errors obtained using only the input features related to melodic and rhythmic content of a piece, thus semantically similar to the information used in the melodic similarity calculation of our method. The rightmost column refers to errors measured after modeling with all features available, thus including score annotations such as dynamic markings, articulations, and slurs. The bottom row corresponds to the results of our method using *k* = 3.

**Table 3:** Performance of note–level algorithms vs. proposed phrase–level method on DS2.

**Algorithm**	**r (34 feat.)**	**MAE (34 feat.)**	**MAE (41 feat.)**
SVM	0.4557	15.44	14.11
ANN	0.3789	22.71	20.08
kNN	0.5910	13.17	12.57
Random forest	0.7319	11.18	10.49
Phrase-level kNN (ours)	0.2956	16.82	—

**Figure 6:**
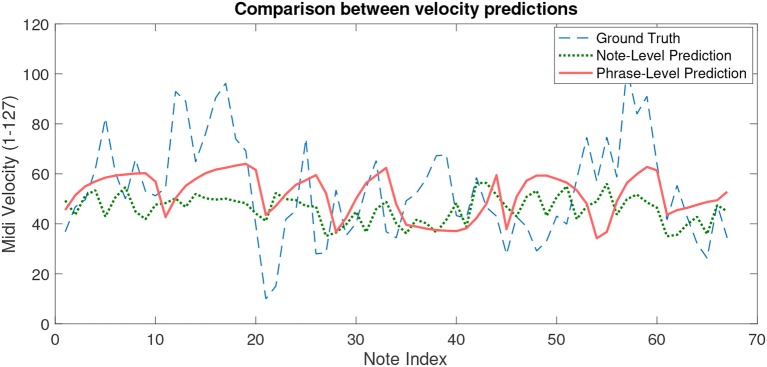
Velocity predictions across notes in a violin piece vs. performed ground truth.

Figure 6 is a plot comparing velocity values from the reference performance, the proposed model predictions, and the best scoring note–level model predictions — both trained with DS2 — for Edward Elgar's *Chanson de Matin, opus* 15, no. 1, bars 2–28.

**Figure 7:**
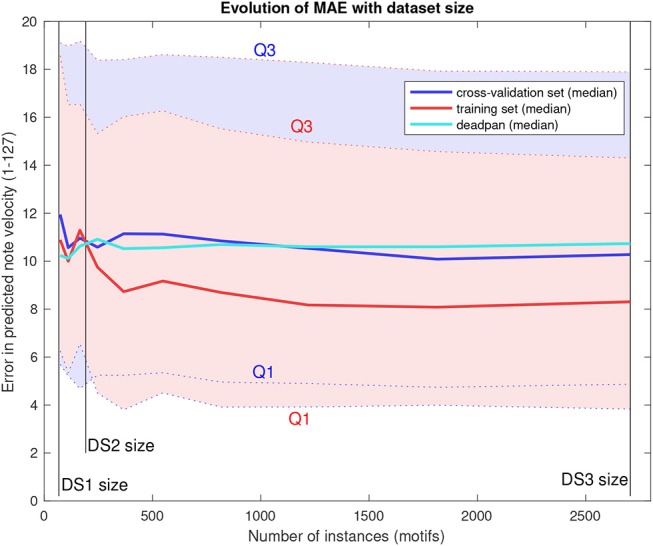
Mean absolute error distribution as a function of dataset size.

Lastly, Figure 7 shows the evolution of mean absolute error distributions in predictions as the training set size increases using the larger DS3 and *k* = 3. The curve for errors in training set predictions are also included to assist interpretation.

### 4.3. Discussion

Three of the four algorithms tested on note–level were able to outperform our phrase–level model in terms of mean absolute errors, and all five exhibit some prediction success if compared to the deadpan MAE of 18.02. Despite the poor rank of our model, these results are encouraging, since they indicate that the majority of the relevant information for predicting expression present in the note features was retained and summarized in the phrase–level form of the dataset.

Visually inspecting the velocity values predicted by the best–performing model, using random forests, against our phrase–level predictions and ground truth values from a recording (Figure 6), it can be seen that the note–level model captures the oscillations in dynamics that happen between adjacent notes whereas the phrase–level model predicts smooth transitions. This partially explains the observed difference in performance, but works in favor of the phrase–level model in our valuation, since in the intended pedagogical application quick transitions are of little use as performance guidelines to students. The appropriate conclusion to draw from these observations, therefore, is that the most adequate ground truth values with which to train a model for such a specific application are not the raw values of dynamics, but rather a smoothed version of them.

Results of the DS3 analysis show that MAE drop for larger datasets but eventually stabilize at a plateau. Though the median error level sits below baseline for large datasets, the large variance (represented by the quartiles indicated as Q1 and Q3) shows precision doesn't improve as much. Using a higher number of neighbors (*k*) only seems to increase the model bias, as we observed higher errors on training set predictions (for *k* = 8, median training set MAE was 9.824 using the entire DS3). These results suggest that the path forward involves the inclusion of more phrase features, and that, for the model's current iteration, increasing training set size further would not be enough to improve its prediction accuracy.

## 5. General Discussion

As we have argued and demonstrated in previous sections, modeling expression in violin performance is a challenging task in many ways. Some examples observed in our first dataset include prolonged, loud notes which we found that sounded harsh in the synthesized version used for perceptual evaluation, but pleasant in the original recording due to the presence of vibrato. We have also met difficulties with notes having very slow attacks in the recordings, for which the placement of a crisp onset in the synthesis inevitably led to rhythmically odd melodies. In many such accounts, participants in the perceptual evaluation rejected the human–based audio samples in favor of the robotic–sounding renditions. Although these findings prevented us from evaluating our modeling strategy as intended, we feel that these results provide a valuable account of the importance of preserving a cohesive set of expressive features as well as the musical context where they appear in order to retain the character in a performance.

Despite not having been able to predict the dynamics or the timing deviations applied by our reference performer, our modeling approach has produced some convincing suggestions of expression, at times worthy of praise by listeners in a blind setting, with considerably less training data than most state–of–the–art models and virtually no time expenditure on model training thanks to the musically coherent approach of processing *motifs* rather than isolated notes. It is also relevant to highlight that even score markings regarding performance such as dynamics and articulation annotations have been ignored by the model, which shows there is still much room for improvement once this knowledge is incorporated into phrase features.

For the desired pedagogical applications, the ability to produce musically valid expressive performances from few examples gives it versatility, allowing students and teachers to select the most relevant reference recordings to make up a training set, for instance for studying the style of a particular performer, or of a specific musical genre. When contrasting the performance of phrase–level against note–level modeling, our phrase–level approach was able to achieve comparable results despite the resulting information compression that comes from summarizing note features in terms of melodic similarity. Additionally, the smoothness inherent in the curves output by our model makes the expressive movements represented by them much easier to follow in real–time by a student.

Perhaps as important as the results concerning our modeling approach are our findings about the methodology of evaluation of expressive performance models for instruments other than piano, for which realistic synthesis is an issue and expression can potentially involve several variables. In those scenarios, we conclude that the modeling is best evaluated if all relevant expressive capabilities offered by the instrument are included in the sound, and preferably modeled as a group to avoid conflicting intentions in the different expressive outputs.

As melodic similarity is central to the expressive engine, expanding the reference violin datasets to include a wide enough variety of melodies, is a natural evolution of this work, in order to investigate in detail the particularities of this instrument when it comes to expression and how much can be improved in the model as a performance predictor.

As briefly mentioned above, there are a number of features, from score markings to the harmonic context of each *motif* that can be incorporated as extra information to the model. Marchini et al. (2014) and Gadermaier et al. (2016) offer good examples of the type of variables that could be considered by our model. Moving toward more complex melody characterization as explored by Gulati et al. (2016) might also provide interesting results.

Lastly, the exploration of different modes of student feedback that can be provided with the outputs of this machine learning model, from auditory to visual to tactile, is an important step to understanding the functions our models should compute as well as being essential to achieving our end goal of improving how people learn and internalize expression in music.

## Ethics Statement

This study was carried out in accordance with the recommendations of the British Psychological Society with written informed consent from all subjects. All subjects gave written informed consent in accordance with the Declaration of Helsinki. The protocol was approved by the Conservatoires UK Research Ethics commitee on 04/04/2017. The consent form presented to subjects read (along with experimental instructions) as follows: Note on Participation and Data Usage Participation in this survey is voluntary and open to anyone aged 16 or older. All responses will be anonymised, and the data collected will be presented at national and international conferences as well as published in academic journals, and may be used for subsequent research. If you decide to take part, by beginning this survey you are providing your informed consent. You will still be free to withdraw at any time. If you are interested to learn more about the results or if you would like your data removed from the project please contact the researchers.

## Author Contributions

FO, SG, and RR: contributed to the design of the model. FO, AP, and RR: designed the evaluation methods. FO and SG: wrote the code for the model and perceptual evaluation. FO analyzed data and wrote this paper.

### Conflict of Interest Statement

The authors declare that the research was conducted in the absence of any commercial or financial relationships that could be construed as a potential conflict of interest.

## Footnotes

^1^https://gist.github.com/fabiozeh

^2^MusicXML 3.1 Specification. The W3C Music Notation Community Group, 2017. https://www.w3.org/2017/12/musicxml31/.

^3^http://musescore.org

^4^https://www.apple.com/lae/logic-pro/

^5^Violin samples from user ldk1609 at freesound.org, licensed with Creative Commons v.1.0 (https://creativecommons.org/licenses/by/1.0/).

^6^http://github.com/fabiozeh/expressiveViolin
